# De novo genome assembly of the soil-borne fungus and tomato pathogen Pyrenochaeta lycopersici

**DOI:** 10.1186/1471-2164-15-313

**Published:** 2014-04-27

**Authors:** Maria Aragona, Andrea Minio, Alberto Ferrarini, Maria Teresa Valente, Paolo Bagnaresi, Luigi Orrù, Paola Tononi, Gianpiero Zamperin, Alessandro Infantino, Giampiero Valè, Luigi Cattivelli, Massimo Delledonne

**Affiliations:** 1 Consiglio per la ricerca e la sperimentazione in agricoltura, Centro di Ricerca per la Patologia vegetale, Via C. G. Bertero 22, 00156 Roma, Italy; 2 Dipartimento di Biotecnologie, Università degli Studi di Verona, Strada le Grazie, 15, 37134 Verona, Italy; 3 Consiglio per la ricerca e la sperimentazione in agricoltura, Centro di Ricerca per la Genomica e la post genomica animale e vegetale, Via S. Protaso 302, 29017 Fiorenzuola d’Arda (PC), Italy; 4 Consiglio per la ricerca e la sperimentazione in agricoltura, Unità di Ricerca per la Risicoltura, S.S. 11 per Torino Km 2,5, 13100 Vercelli, Italy

**Keywords:** *Pyrenochaeta lycopersici*, Pathogenicity, Genome assembly, Next Generation Sequencing technologies (NGS)

## Abstract

**Background:**

*Pyrenochaeta lycopersici* is a soil-dwelling ascomycete pathogen that causes corky root rot disease in tomato (*Solanum lycopersicum*) and other *Solanaceous* crops, reducing fruit yields by up to 75%. Fungal pathogens that infect roots receive less attention than those infecting the aerial parts of crops despite their significant impact on plant growth and fruit production.

**Results:**

We assembled a 54.9Mb *P. lycopersici* draft genome sequence based on Illumina short reads, and annotated approximately 17,000 genes. The *P. lycopersici* genome is closely related to hemibiotrophs and necrotrophs, in agreement with the phenotypic characteristics of the fungus and its lifestyle. Several gene families related to host–pathogen interactions are strongly represented, including those responsible for nutrient absorption, the detoxification of fungicides and plant cell wall degradation, the latter confirming that much of the genome is devoted to the pathogenic activity of the fungus. We did not find a MAT gene, which is consistent with the classification of *P. lycopersici* as an imperfect fungus, but we observed a significant expansion of the gene families associated with heterokaryon incompatibility (HI).

**Conclusions:**

The *P. lycopersici* draft genome sequence provided insight into the molecular and genetic basis of the fungal lifestyle, characterizing previously unknown pathogenic behaviors and defining strategies that allow this asexual fungus to increase genetic diversity and to acquire new pathogenic traits.

## Background

**Figure 1:**
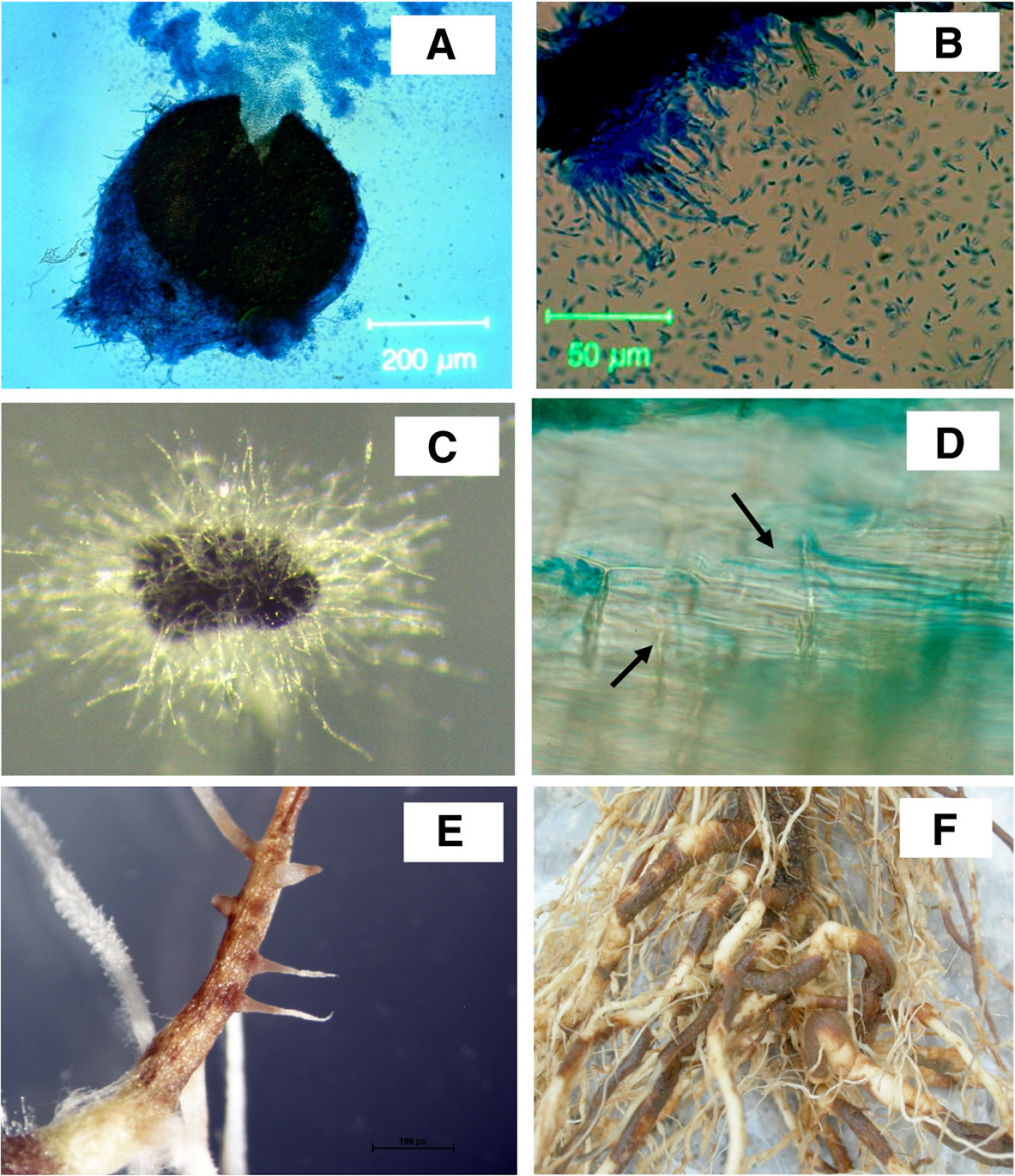
**Infection of tomato roots by *P. lycopersici. *(A)** Light micrograph of a pycnidium with erupting conidia. **(B)** Light micrograph of conidiophores with conidia. **(C)** Stereomicrograph of a *P. lycopersici* microsclerotium germinating on artificial media. **(D)***P. lycopersici* hyphae (black arrows) transformed with GUS + growing within young tomato roots. **(E)** Symptoms caused by *P. lycopersici* growing on young tomato rootlets artificially infected with the fungus. **(F)** Naturally-infected tomato roots with extensive corky root rot symptoms.

*Pyrenochaeta lycopersici* is the soil-borne fungal pathogen responsible for corky root rot (CRR) disease in tomato [1,2]. The fungus also infects other *Solanaceous* species including pepper, eggplant and tobacco, and other cultivated crops such as melon, cucumber, spinach and safflower [3-5]. The pathogen causes significant yield losses in tomato crops, both in the greenhouse and in the field, in many tomato-growing areas of the world. The majority of commercial varieties are susceptible to *P. lycopersici* and sources of partial genetic resistances occur only in wild tomato species [6]. The use of susceptible cultivars combined with continuous cropping and the lack of effective soil treatments has encouraged the rapid spread of CRR disease resulting in fruit yield losses of up to 75% [7,8]. The sequencing of 18S nrDNA (SSU) and 28S nrDNA (LSU) indicate that *P. lycopersici* is an ascomycete in the order *Pleosporales,* along with other necrotrophic and hemibiotrophic plant pathogens representing genera such as *Cochliobolus, Pyrenophora, Phaeosphaeria, Leptosphaeria, Pleospora, Phoma* and *Didymella*[9]*.* A low grade of genetic variability was shown within *P. lycopersici* isolates, when investigated by RAPD and RFLP analysis [10], and the use of ISSR and AFLP markers [11,12].Little is known about the biology, life cycle and infection structures of this species. A telomorph has not been described and even the anamorph is rare in nature. The latter is characterized by pycnidia containing solitary, mostly branched conidiophores bearing hyaline unicellular conidia (Figure 1A, B). They have never been found on infected tissues in nature and sporulation is difficult to induce in culture. *P. lycopersici* produces microsclerotia on host plant roots and in artificial medium (Figure 1C), and these are the overwintering structures and primary infective propagules in the soil, remaining dormant but viable for at least 15 years [3,13,14]. Under favorable conditions, hyphae germinate from the sclerotia and asymptomatically infect the epidermal cells of host roots (Figure 1D). Approximately 48h after initial penetration, the infected host cells die and secondary hyphae develop within them, a stage associated with the appearance of disease symptoms such as tissue browning and necrosis (Figure 1E). The invasion of living host cells by primary hyphae continues at the expanding margins of the lesion, surrounding a central zone of dead cells. *P. lycopersici* is generally recognized as a hemibiotroph, with biotrophy and necrotrophy probably occurring simultaneously during the later stages of infection, as the necrotic lesions expand and the cells walls are degraded by lytic enzymes [15-17]. Ultimately, the main root is entirely colonized causing the typical corky root lesions, whereas secondary roots often escape infection (Figure 1F) [18]. Only a few *P. lycopersici* sequences (mostly derived from ITS regions) are deposited in the NCBI database, and little is known about the molecular mechanisms involved in *P. lycopersici* pathogenicity/virulence and host–pathogen interactions. Recently, a cDNA-AFLP based transcriptomic approach was used to monitor the expression of plant and pathogen genes during a compatible interaction between *P. lycopersici* and tomato [19,20]. This allowed the subsequent isolation and characterization of a *P. lycopersici* endoglucanase which is strongly induced during the infection of tomato roots and whose expression is positively correlated with disease progression [17]. A secreted pathogenicity factor that induces cell death during the penetration of tomato roots has also been identified [21]. Here we report the *de novo* assembly of the *P. lycopersici* genome based on Illumina sequencing and the functional characterization of the draft sequence by integrating RNA-Seq data, followed by an in-depth analysis of the virulence mechanisms and potential pathogenicity effectors encoded by this soil-transmitted pathogen.

## Results

### Genome sequencing and assembly

Genomic DNA obtained from the virulent *P. lycopersici* isolate CRA-PAV_ER 1211 was sequenced using Illumina 100-bp paired-end reads. Two libraries were prepared with mean fragment sizes of 460 and 560 bp respectively and sequenced obtaining 85 millions of fragments for a total of 17Gb, corresponding to approximately 300-fold coverage of the final assembly.

The *P. lycopersici* genome was assembled *de novo* using a de Bruijn graph-based (DBG) assembly strategy with a k-mer of 55 (Additional file 1: Figure S1) before reassembly using the overlap-layout-consensus (OLC) method. We chose to use this strategy to take advantage of both the higher efficiency of DBG method in assembling millions of reads and the higher sensitivity of OLC method [22,23]. In fact, the OLC re-assembly of sequences previously assembled with DBG method reduced the total number of sequences by a 40% (from 11,617 to 7,079) while increasing the average contig length by a 60% (from 4,834 to 7,747 bp) and not reducing significantly the total number of assembled bases (54.8 Mbps vs. 56.2 Mbps).

The assembly statistics are summarized in Table 1, comprising 7,079 contigs with an N50 of 73.4 kb for a total sequence of 54.9 Mb. The comprehensiveness of the gene space covered by the assembly and annotation procedures was assessed by screening for 248 core eukaryotic genes (CEGs) [24] revealing hits for 238 CEGs (95.97%) with complete match and 241 with partial match (97.18) (Additional file 2: Table S1).

**Table 1:** ***Pyrenochaeta lycopersici *** **genome statistics**

	**Scaffolds**	**Contigs**
Number of sequences	7,079	18,757
Maximum sequence length (bp)	768,125	440,042
Average length (bp)	7,747	2,856
N50 (bp)	73,363	33,727
N90 (bp)	313,035	127,052
***Sequences > 500bp***	**Scaffolds**	**Contigs**
Number of sequences	5,176	10,176
Average length (bp)	10,479	5,037
N50 (bp)	75,634	37,929
N90 (bp)	315,231	10,146
***Sequences > 1Kb***	**Scaffolds**	**Contigs**
Number of sequences	4,056	5,863
Average length (bp)	13,174	8,217
N50 (bp)	76,086	43,602
N90 (bp)	315,231	130,015
***Sequences > 5Kb***	**Scaffolds**	**Contigs**
Number of sequences	1,530	1,350
Average length (bp)	30,926	29,077
N50 (bp)	96,502	55,291
N90 (bp)	369,997	139,267
***Sequences > 10Kb***	**Scaffolds**	**Contigs**
Number of sequences	784	858
Average length (bp)	53,680	41,789
N50 (bp)	112,715	62,806
N90 (bp)	391,898	139,822
Total number of assembled bases (without Ns)	54,841,665 bp (53,572,698 bp)	
Estimated x-fold genome coverage	217×	
GC percentage content	39.40%	
Number of annotated gene loci	17,411	
Number of annotated putative transcripts	27,275	
Mean gene size (bp)	2,015	
Mean exon size (bp)	676.6	
Mean number of exons per gene	4.1	

### Gene annotation

Genes were annotated by a combination of *ab initio* prediction [25] followed by the reference annotation-based transcript (RABT) assembly of sequences obtained from the RNA-Seq analysis of two *P. lycopersici* samples grown *in vitro* and during interaction with tomato cv Moneymaker, respectively [26]. We annotated 17,411 genes with 27,275 transcripts, among which 9,553 loci were detected by both approaches and 2,797 only by the analysis of RNA-Seq data (Table 1). Thus, while the number of genes might be slightly overestimated because of *ab initio* prediction limitations, at least 70.8% of the annotations were supported by experimental evidences. We confirmed that the assembly was able to capture full-length genes by searching the predictions for full open reading frames (ORFs), finding that most of the genes (80.76%) contained start and stop codons. The RNA-Seq data were then assembled *de novo* and the resulting contigs were mapped onto the draft genome. From the 31,746,550 reads, we obtained 27,982 putative transcripts, 27,574 (98.5%) of which could be mapped onto the assembled genome further validating the comprehensiveness of the gene space represented.

Many of the identified transcripts (75.8%) were conserved in other species as shown by hits against sequences in the NCBI NR protein database (e-value < 1E-0.6) and the Uniprot SwissProt Fungi protein database (e-value < 1E-0.7). This analysis showed that the most represented species were fungal pathogens and the top four species were phytopathogenic fungi (Additional file 1: Figure S2). Based on the identity of the most similar proteins, at least one Gene Ontology term was assigned to 8,507 transcripts. The most represented functional categories are summarized in Additional file 1: Figure S3. We found 3,962 orphan genes (22.8%) with no matches against known proteins or protein domains [27], which is similar to the proportion of orphan genes found in *Sordaria macrospora* (22%) [28] and *Macrophomina phaseolina* (29%) [29]. RNA-Seq analysis showed that 1,936 of the 3,962 orphan genes were transcribed and were therefore likely to be functional.

### Phylogenetic relationships

**Figure 2:**
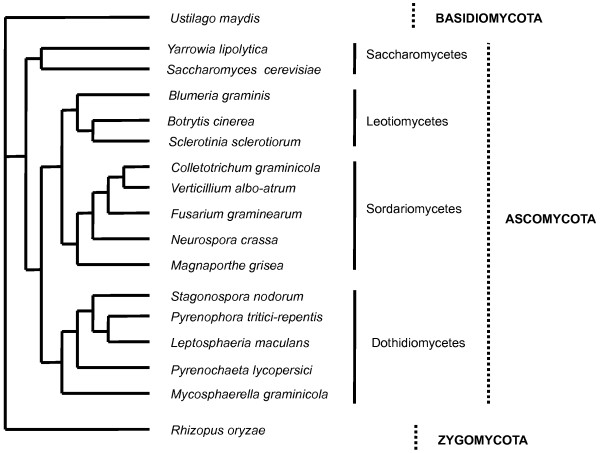
**Cladogram showing the phylogenetic relationship between *P. lycopersici *and 16 other fungi with sequenced genomes.** The unscaled tree was built based on comparison of whole genome sequences of 16 fungi using *Rhizopus oryzae* as outgroup.

The phylogenetic analysis based on whole genome sequences of *P. lycopersici* and 16 other fungal species with diverse lifestyles (from necrotrophs to biotrophs) is shown in Figure 2. This confirmed that *P. lycopersici* belongs to the class Dothideomycetes and the order Pleosporales. A neighbor-joining phylogenetic tree, using *Rhizopus oryzae* as the outgroup, revealed four major clusters representing *Saccharomycetes, Leotiomycetes, Sordariomycetes* and *Dothideomycetes. P. lycopersici* appeared to be more closely related to hemibiotrophic and necrotrophic plant pathogens of the genera *Leptosphaeria, Pyrenophora* and *Stagonospora* than to biotrophs such as the genera *Blumeria* and *Ustilago*.

We performed a pairwise comparison between *P. lycopersici* and *Fusarium oxysporum f. sp. lycopersici* genomes to assess the genomic distribution of similarity regions. We choose *F. oxysporum* among the top ranked species (Additional file 2: Table S2) as a complete genome anchored to chromosomes was available for this fungus. As expected the analysis showed regions of similarity at aminoacidic level throughout all the *F. oxysporum* chromosomes with the exception of the four *F. oxysporum* dispensable chromosomes 3, 6, 14 and 15 (Additional file 1: Figure S4).

### Vegetative incompatibility

**Figure 3:**
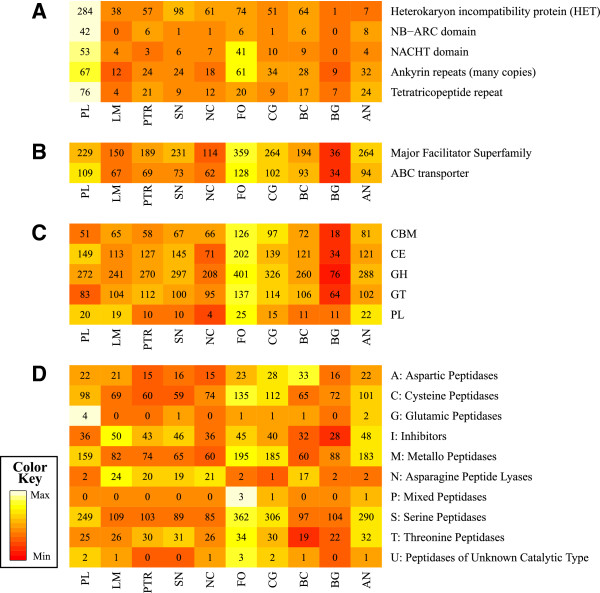
**Comparison of repertoires of important fungal protein families.** The heat maps compare the number of functional domains identified in *P. lycopersici* (PL) and nine other fungal pathogens: *Aspergillus nidulans* (AN), *Botrytis cinerea* (BC), *Blumeria graminis* (BG), *Colletotrichum graminicola* (CG), *Fusarium oxysporum* (FO), *Leptosphaeria maculans* (LM), *Neurospora crassa* (NC), *Pyrenophora tritici-repentis* (PTR) and *Stagonospora nodorum* (SN). **A)** Vegetative incompatibility-related domains. **B)** Virulence-related efflux pump domains. **C)** Carbohydrate-active enzymes (CAZymes), GH = glycoside hydrolases; GT = glycosyltransferases; PL = polysaccharide lyases; CE = carbohydrate esterases; CMB = carbohydrate-binding modules. **D)** Peptidases by superfamily.

Fungi can propagate both by sexual and vegetative reproduction. The latter is controlled by approximately 50 heterokaryon incompatibility (HET) modules in most pathogenic fungi, whereas there was evidence of 284 modules in *P. lycopersici* (Figure 3a; Additional file 2: Table S4). Accounting for a possible overestimation of the total number of genes by a 40%, based on the percentage of annotations not supported by RNASeq data, the HET protein modules are anyway expanded by 3.9 times, in average, compared to other fungi. Other proteins that are functionally associated with HET contain NTPase, NB-ARC and NACHT domains, which are involved in the regulation of the immune response and are related to apoptosis/programmed cell death in animals and fungi [30]. We identified 53 NACHT proteins encoded by the *P. lycopersici* genome, which is slightly more than the number in *F. oxysporum* (41) and much higher than in other fungi, which range from 0 in *Blumeria graminis* to 10 in *Colletotrichum graminicola*. The *P. lycopersici* genome also encoded a significantly greater number of proteins containing ankyrin (ANK) and tetratricopeptide repeats (TPR), which mediate protein-protein interactions among HET proteins. Altogether, the number of HI related proteins includes 522 domains, e.g. more than double the number encoded by the imperfect fungal pathogen *F. oxysporum*. About 75% of the modules for all the families related to vegetative incompatibility were supported by RNASeq expression data.

### Pathogenesis related genes

We screened the *P. lycopersici* genome sequence against Phi-base, a database that collects pathogenicity, virulence and effector genes from fungi, oomycetes and bacterial pathogens. This revealed that 2,196 (12.6%) of the *P. lycopersici* genes were homologous to putative pathogenicity genes (Additional file 3). Comparative analysis with nine sequenced filamentous fungi showed that the gene families with the largest number of shared pathogenicity genes were heterokaryon incompatibility proteins (284), glycoside hydrolase proteins (272), major facilitator superfamily (MFS) type membrane transporters (229), fungal transcription factors (174), protein kinases (170) and cytochrome P450 (125) (Table 2).

**Table 2:** Highly represented protein families

	**AN**	**BC**	**BG**	**CG**	**FO**	**LM**	**NC**	**PL**	**PTR**	**SN**	P value*	Adjusted P-value**
**Genome assembly size (Mb)**	30.0	42.7	87.9	51.6	61.4	44.9	41.0	54.8	37.8	37.3		
**Number of genes**	10,827	16,714	7,073	12,361	21,354	12,469	9,730	17,411	12,300	12,382		
**Ankyrin repeats**	32	28	9	34	61	12	18	67	24	24	*8.90E-04*	*5.14E-04*
**D-Tyr-tRNA(Tyr) deacylase**	1	1	1	1	1	1	1	5	1	1	NA	NA
**DDE superfamily endonuclease**	4	12	2	2	13	2	0	89	11	1	*1.70E-08*	*1.47E-07*
**Double stranded RNA binding domain**	1	2	2	2	2	2	2	7	2	0	*1.61E-06*	*2.32E-06*
**Mo25-like**	1	1	1	1	1	1	1	5	1	1	NA	NA
**NACHT domain**	4	9	0	10	41	4	7	53	3	6	*1.90E-04*	*1.24E-04*
**NAD-specific glutamate dehydrogenase**	0	0	0	0	0	0	0	8	0	0	NA	NA
**NB-ARC domain**	8	6	0	1	6	0	1	42	6	1	*3.69E-08*	*1.60E-07*
**NPL4 family**	1	1	1	1	1	1	1	5	1	1	NA	NA
**Tetratricopeptide repeat**	24	17	7	9	20	4	12	76	21	9	*2.82E-07*	*8.14E-07*
**Transmembrane alpha-helix domain**	2	1	0	3	3	1	0	10	4	0	*9.52E-06*	*9.15E-06*
**Transposase IS4**	0	0	0	1	17	0	0	11	0	0	*1.28E-02*	*5.28E-03*
**hAT family dimerisation domain**	0	1	0	0	76	0	0	30	0	0	1.30E-01	*4.17E-02*
**Helix-turn-helix, Psq domain**	0	8	0	0	0	0	0	24	0	0	*1.01E-06*	*1.75E-06*
**Major facilitator superfamily**	264	194	36	264	359	150	114	229	189	231	5.21E-01	1.45E-01
**Cytochrome P450**	116	120	13	138	157	62	40	125	102	122	2.63E-01	*7.68E-02*
**Fungal specific transcription factor domain**	232	94	20	176	252	100	77	174	96	100	*9.93E-02*	*3.31E-02*
**Protein kinase domain**	105	94	82	104	146	116	103	170	108	115	*2.00E-04*	*1.24E-04*
**Heterokaryon incompatibility protein (HET)**	7	64	1	51	74	38	61	284	57	98	*1.01E-06*	*1.75E-06*

Selected protein families highly represented in *P. lycopersici* genome. AN, *Aspergillus nidulans*; BC, *Botrytis cinerea*; BG, *Blumeria graminis*; CG, *Colletotrichum graminicola*; FO, *Fusarium oxysporum*; LM, *Leptosphaeria maculans*; NC, *Neurospora crassa*; PL *Pyrenochaeta lycopersici*; PTR, *Pyrenophora tritici repentis*; SN, *Stagonospora nodorum*. *The cutoff of significance is set at P < 0.05. **Benjamini and Hockberg method was used to calculate adjusted p-values [90].

The ATP-binding cassette (ABC) transporters and MFS-type membrane transporters were largely represented in the *P. lycopersici* genome, comprising 109 and 229 modules respectively (Figure 3b and Additional file 2: Table S2). Transcriptome analysis showed that 85% of ABC modules and 75% of MFS-type membrane transporters belonged to transcripts expressed. The *P. lycopersici* genome also encoded a large number (125) of cytochrome P450 proteins, as shown for other necrotrophic and hemibiotrophic pathogens such as *B. cinerea* (120), *C. graminicola* (138), *F. oxysporum* (157) and *S. nodorum* (122).

The *P. lycopersici* predicted genes also included 597 sequences matching 94 different subfamilies of peptidases (Additional file 2: Table S3). Almost all known peptidase families were represented in the *P. lycopersici* transcriptome, with DmpA aminopeptidase 1 (P1) as the only exception. The most represented clans were the serine peptidase (S clan) and metallopeptidase (M clan), a common feature of fungal pathogens. The comparative analysis of gene families and PFAM domains with several other fungi showed that the number of peptidases in *P. lycopersici* is comparable to other hemibiotrophic pathogens as *C. graminicola* and *F. oxysporum*. Metallopeptidase group of aminopeptidases (M1, M18, M24, M28) were highly represented, with 43 domains in *P. lycopersici* (shown in yellow in Additional file 2: Table S3) compared to 34 and 30 of *C. graminicola* and *F. oxysporum* respectively. Analysis of expression data confirmed that 36 of this modules (85%) corresponded to expressed transcripts. Other metallopeptidase gene families had also undergone expansion in the *P. lycopersici* genome, including pappalysin, Ste24 and deubiquitinating peptidase (shown in green in Additional file 2: Table S3). A summary is reported in Figure 3d.

### Genes involved in carbohydrate degradation (CAZymes)

Carbohydrate degradation is an important component of fungal pathogenicity and virulence, so we examined the *P. lycopersici* CAZome in detail compared with nine other fungi with complete genome sequences (Additional file 2: Table S6). The *P. lycopersici* genome encodes 575 CAZyme modules, including 272 glycoside hydrolases, 83 glycosyltransferases, 20 polysaccharide lyases, 149 carbohydrate esterases and 51 carbohydrate-binding modules (CMBs). CE1 and CE10 families of carbohydrate esterases including acetyl xylan esterase (EC 3.1.1.72), cinnamoyl esterase (EC 3.1.1), feruloyl esterase (EC 3.1.1.73), carboxylesterase (EC 3.1.1.1), S-formylglutathione hydrolase (EC 3.1.2.12) and sterol esterases are particularly represented (53 CE1 modules out of which 77% are detected as expressed by RNASeq analysis, 54 modules for CE10 family 73,5% of which are expressed) similarly to other hemibiotrophs such as *C. graminicola* and *F. oxysporum* (Additional file 2: Table S6). Cellulose-degrading enzymes were also well represented in *P. lycopersici*, including seven cellobiohydrolases (GH6), ten β-1,3-glucanases (GH55) and eight GH105 glycoside hydrolases (unknown mechanism). Finally, the complex of carbohydrate cleaving enzymes was completed by 20 genes encoding polysaccharide lyases, including 13 encoding PL3.

## Discussion

We report here the first genome analysis of a *Pyrenochaeta* species, the soil-borne filamentous fungus of ascomycete clade *Pyrenochaeta lycopersici*, which is the etiological agent of tomato corky root rot. The *P. lycopersici* genome assembly shows that a genome reconstruction approach based uniquely on paired-end Illumina reads is highly effective in reconstructing contigs containing almost full length genes. To validate the genome assembly we assessed the completeness of the gene space represented and we found that, in fact, most of the core eukaryotic conserved genes and the transcripts reconstructed from RNA-Seq data of fungus grown *in vitro* and during interaction with the host were represented on the assembled genome. Based on published assemblies for the phytopathogens *L. maculans*, *P. teres*, *P. nodorum* and *P. tritici-repentis* the number of annotated genes in *P. lycopersici* is very similar (17,411 versus 12,469, 11,799, 12,382 and 12,300 respectively) [31-34]. Most of the gene annotations are supported by the presence of a full ORF (about 80%) and a large fraction of them is validated by RNA-Seq data (70%), thus representing an high quality resource for the study of functions encoded by *P. lycopersici* genome. It’s worth noting that we also identified more than 2,700 genes supported by transcriptomic data but not detected by the prediction algorithm. This is not completely surprising as, even if properly trained, a prediction software will not predict all the genes of an eukaryote organism. An hybrid annotation approach, based on integration of gene predictions and massive parallel sequencing of transcripts, is thus needed to perform a comprehensive annotation of genes in a fungal genome. Overall these data confirm the high quality of the assembly and annotation obtained particularly in terms of the completeness and quality of the gene catalog represented.

The *P. lycopersici* assembly produced constitutes an invaluable support to understand the unique phenotypical features of this pathogen and allow to investigate the molecular basis of the reproductive behavior and of the mechanisms involved in the pathogenesis. Sexual mating of *P. lycopersici* has never been observed in nature, leading to speculation regarding its reproductive cycle. In agreement with its reproductive behavior based on generating spores (conidia) by mitosis, *P. lycopersici* apparently lacks of mating-type (*MAT*) genes that control the choice between sexual and asexual reproduction, suggesting the species is incapable of sexual reproduction. A potential alternative source of genetic variation in *P. lycopersici* is vegetative hyphal fusion controlled by *HET* genes, allowing horizontal gene or chromosome transfer potentially followed by non-meiotic recombination. When individuals with the same *HET* genotype meet, they can produce a viable heterokaryon by anastomosis, whereas individuals with different *HET* genotypes form a fusion cell which is compartmentalized and undergoes a form of programmed cell death termed vegetative or heterokaryon incompatibility [35]. Although the significance of heterokaryon incompatibility responses is poorly understood [36], it may limit the transmission of mycoviruses and other deleterious replicons between strains [37]. Strong selective pressure is likely to be responsible for the extensive amplification of *HET* genes and genes with related functions in *P. lycopersici,* suggesting that *HET* genes play a key role in the transfer of genetic information in this species, and that a strict regulation of the vegetative reproduction is important for the generation of the variability necessary for the adaptation to the environment and to host defense mechanisms.

*From a pathogenetic perspective P. lycopersici* is considered a hemibiotrophic fungus because cell wall hydrolytic activity is not detected during the initial infection of tomato plants and few symptoms are visible, whereas later infection involves the secretion of cell wall degrading enzymes that cause the root to collapse [15,17]. This was confirmed by our data by the comparison of *P. lycopersici* sequences with those of other fungi which showed a clear phylogenetic relationship with hemibiotrophic and necrotrophic plant pathogens. Moreover, the analysis of gene functions and comparison of gene sequences with those of other fungi showed that a large fraction of genome is devoted to pathogenetic activity and showed a large overlap of the gene inventory to that of other plant pathogens such as *L. maculans*, *P. teres*, *P. nodorum* and *P. tritici-repentis*.

The first and major barrier to infection by fungal pathogens in plants is the cell wall and cellulose is the main component of plant biomass. Phytopathogenic fungi therefore secrete a cocktail of hydrolytic enzymes known as carbohydrate-active enzymes (CAZymes), which are required to penetrate and then degrade the cuticle and cell wall [38]. The analysis of CAZome of *P. lycopersici* showed a stronger resemblance to that of hemibiotrophic and necrotrophic plant pathogens such as *C. graminicola, L. maculans* and *P. tritici-repentis* than to that of biotrophs such as *B. graminis.* In fact, the *P. lycopersici* genome encoded a large number of cellulose-degrading enzymes, as glycoside hydrolases, required for the complete breakdown of the plant cell wall for successful infection. In particular, the CE1 and CE10 families of carbohydrate esterases, which are required to degrade hemicellulose and thus facilitate the complete hydrolysis of polysaccharides in the cell walls of a wide range of plant species, as well as pectate-lyases (which doubles the number of *PL*s in *P. lycopersici* compared to other fungi) were largely represented. This may reflect in an enhanced activity in cleaving pectic polymers, which are more abundant in dicotyledonous plants. *Among the glycoside hydrolases,* the GH61 family carries out cellulose hydrolysis using a synergic mechanism in concert with canonical cellulases [39,40]. The expansion of the GH61 family in *P. lycopersici* mirrors the expansion of this family in the order *Pleosporales*[41]. The GH61 gene family is exclusive to fungi and structure–function analysis of some enzymes belonging to this class, showed they cleave cellulose using an oxidative mechanism, and therefore they are not canonical glycoside hydrolases. The new biochemical mechanism proposed for GH61 proteins redefined this class of enzymes as polysaccharide monooxygenases (PMOs) [42,43]. A *P. lycopersici* endo-β-1,4-glucanase gene (*Plegl1*) from the GH61 family is expressed in a manner that corresponds to disease progression [17,44]. *Plegl1* is thus far the only gene known to be induced in a fungal phytopathogen during infection, suggesting a role in pathogenesis. These data suggest the hypothesis that *P. lycopersici* might have evolved diverse strategies for cellulose digestion.

Other proteins which play an important role in the interaction with the host by detoxifying plant defense compounds are cytochrome P450 proteins. *P. lycopersici*, similarly to other hemibiotroph and necrotroph fungal pathogens, shows an high number and an high variety of genes coding for this protein family.

Peptidases are pathogenicity-related enzymes that are secreted to facilitate penetration and colonization of the host by degrading the plant cell wall [45-48] and are required for the degradation of plant defense proteins [49-51]. The *P. lycopersici* genome encodes a large number of metallopeptidases, covering 10 major peptidase families and 94 subfamilies, in agreement with the general properties of Dothideomycetes genomes, mostly representing hemibiotrophs [41]. The comparison of 18 genomes revealed that Dothideomycetes have a wider range of exopeptidases and endopeptidases than other fungal phytopathogens, including the greatest number of secreted metallopeptidases, but fewer aspartic peptidases (A01) than necrotrophs, saprotrophs and ectomycorrhizal symbionts [41]. The aminopeptidase gene family is the most highly represented among all the metallopeptidase families represented in the *P. lycopersici* genome and it is well documented that bacteria and fungi which secrete aminoproteases are generally pathogenic [52,53].

Another class of proteins which play an important role during host invasion is that of transporters. Transporters import nutrients and export secondary metabolites produced by the fungal pathogens as virulence factors but have a role also in removing toxic compounds. In particular, ABC and MFS transporters are the major families involved [54-56] as they are required to export host-specific toxins (HSTs) and mycotoxins [57-59], remove inhibitory defense compounds such as phytoalexins produced by the host plant [60], and confer resistance to fungicides [61]. Although the virulence of the fungus is not strictly dependent on the abundance of these transporter families [54] is notable that the ABC transporter family in *P. lycopersici,* together with *F. oxysporum,* is the most diverse and abundant compared to all the other fungi, and is therefore likely to be intimately associated with the virulence mechanism. Overall the analysis of *P. lycopersici* genome clearly shows that this pathogen evolved its pathogenetic mechanisms through an expansion both of genes involved in the penetration and degradation of the host tissues and by the expansions of gene families necessary to counteract the defense mechanisms of the host.

## Conclusions

*P. lycopersici* genome reveals a significative expansion of specific genes families related both to pathogenesis and to reproduction mechanisms, which suggests that *P. lycopersici* has undergone to a specialization and adaptation process during its evolution. The assembly presented constitutes an important resource to understand the molecular bases of corky root rot and more in general to enrich current knowledge of plant-pathogen interaction mechanisms.

## Methods

### Sample and library preparation

Genomic DNA was isolated from a virulent *P. lycopersici* isolate (CRA-PAV_ER 1211) grown on Potato Dextrose Agar (PDA) medium, by the method described by Cenis as previously described [62] with the following modifications: 200 mg of mycelium was frozen in liquid nitrogen, pulverized, and incubated in 300 μl of lysis buffer (200 mM Tris-HCl pH 8.5, 250 mM NaCl, 25 mM EDTA, 0.5% SDS) for 10 min at 65°C. We then added 150 μl 3 M sodium acetate (pH 5. 2), incubated at –20°C for 10 min and centrifuged at 10000 × g for 30 min. The supernatant was transferred in a fresh tube and the DNA was precipitated by adding an equal volume of isopropanol, centrifuging at 10000 × g for 10 min and washing with 70% ethanol. Finally the DNA was purified using the NucleoSpin Extract II kit (Macherey-Nagel, Düren, Germany). Total RNA from vegetative mycelium grown on PDA medium was extracted using the RNeasy Midi kit (Qiagen, Hilden, Germany). Total RNA from infected tomato roots of cv Moneymaker was extracted using the NucleoSpin RNA Plant 2 kit (Macherey-Nagel) after 8 days post infection (dpi).

Genomic DNA (6 μg) was fragmented by nebulization at 35 psi for 6 min. DNA libraries with insert sizes of 400 bp and 560 bp were prepared from 1 μg of fragmented genomic DNA using the paired-end TruSeq DNA Sample Preparation Kit (Illumina Inc., San Diego, CA, USA). The library quality was determined using the High Sensitivity DNA Kit (Agilent, Wokingham, UK).

Total RNA samples were assessed for quality using an RNA 6000 Nano Kit (Agilent) and 2.5-μg aliquots were used to isolate poly(A) mRNA for the preparation of a non-directional Illumina RNA-Seq library using the TruSeq RNA Sample Prep Kit (Illumina). The quality of the library was checked using the High Sensitivity DNA Kit (Agilent).

### Sequencing and data preprocessing

Libraries were sequenced with an Illumina GAIIx sequencer generating 100-bp paired-end sequences for DNA libraries and 130-bp paired-end sequences for RNA libraries.

The sequences were pre-processed by removing reads with a number of N >10 or with a read quality <20 using a custom script. Adapters were clipped using Scythe v0.980 [63] and bases on both 3′ ends with a quality <20 were trimmed using Sickle v0.940 [64], eventually entirely removing the fragment if the length was reduced to < 50 bp.

### De novo assembly and gene catalog assessment

The genome was assembled *de novo* using Velvet v1.1.06 [65] with the following parameters: –exp_cov auto (automatic calculation of expected coverage), –scaffolding (scaffolding of contigs with paired-end reads) and –min_contig_lgth 200 (mimimun contig length = 200). The optimal k-mer length was determined by adjusting the k-mer length from 39 to 67 bp in 4-bp increments and using the k-mer for which the N50 and the maximum contig length reached the highest value (Additional file 1: Figure S1). The resulting contigs were then re-assembled with CAP3 v10/15/07 [66] using standard parameters.

Core Eukaryotic Genes (CEGs) were aligned with assembled genome using BLAST and hits were considered significant when the sequence identity was >65%.

RNA-seq reads from *in vitro* mycelia were assembled using Trinity v r2011-11-26 [67] with standard parameters, jaccard clip on and a minimum contig length of 200 bp. Assembled contigs were mapped using GMAP [68] with standard parameters.

### Gene annotation

The final assembly was processed by GeneMark.hmm-ES v2.3e [25] with standard parameters and no *a priori* information. The genome was masked for repetitive and low-complexity regions with RepeatMasker v open-3.3.0 [69] with standard parameters and a general repeats database. The resulting annotation was refined using TopHat v1.4.1 and Cufflinks v1.2.1 [70] on the two RNA-seq libraries in RABT mode with standard parameters. ORFs were identified for each transcript using CPC v0.9.r2 [71].

### Phylogenetic analysis

A phylogenetic tree based on the comparison of whole genomes of *P. lycopersici* and 16 other fungi was constructed using CVTree v2 [72] at a k-mer length of 7. *Rhizopus oryzae* was used as the outgroup for building the unscaled tree [73]. The *P. tritici-repentis* and *C. graminicola* proteomes were obtained from the *Colletotrichum* Sequencing Project [74], the *L. maculans* proteome was obtained from the *L. maculans* genome project [75], and the *B. graminis* proteome was obtained from the *Blumeria* sequencing project [76]. Unless otherwise stated, the remaining proteomes were obtained from the CVtree inbuilt genome database [77].

### Functional annotation

Functional annotation was initiated by using each sequence as a BLAST query [78] against the NCBI Non Redundant database retrieved 2012-09-14 [79] (e-value < 1E-0.6) and the Uniprot SwissProt Fungi protein database retrieved 2012-04-30 [80] (e-value < 1E-0.7). The results were analyzed using Blast2GO [81] and integrated with InterPro results [82]. Conserved protein domains in *P. lycopersici* and all the other fungi considered (AN, BC, BG, CG, FO, LM, NC, PT-R, SN) were identified using HMMer v3.0 [83] to identify homology with proteins in the Pfam-A database (v26.0, 2011-11) [84]. Sequence conservation was considered significant at an e-value threshold <1e-6 for both the entire sequence match and for the independent E-value of the single domain match. CAZymes (v2.0) [85] homology was also inspected using HMMer. Alignments were considered significant at an alignment length > 80 residues, E-value < 1e-5 and HMM profile coverage > 30% or alignment length < 80 residues and E-value < 1e-3 and HMM profile coverage > 30%. BLASTX was used to identify sequences homologous to known pathogenic genes (PHI-base ver 3.2) [86], peptidases (MEROPS ver 9.8) [87], zinc fingers (C2H2 ZNF db, ver. 2007-10-03) [88], MAP kinase sequences from NCBI NR (retrieved 2013-01-30) [79] and membrane transport proteins (TCDB, ver. 2011-July-15) [89] (e-value <1e-10). Sequences with significant hits against membrane transport proteins were also used as BLASTX queries against a G protein-coupled receptors database (GPCRDB, retrieved 2013-01-30) [90] and fungi major facilitator superfamily sequences from NCBI NR [79].

Significance of protein families abundance differences between *P. lycopersici* and all the other fungal plant pathogens (BC, BG, CG, FO, LM, PT-R, SN) was assessed by a 1-sample t-test. Variance was estimated based on protein families abundance data of all the fungal pathogens taken into account (*P. lycopersici* excluded). P-values were corrected according to Benjamini and Hochberg [91] on the full dataset of comparisons.

Genome coverage has been estimated by mapping the reads on the assembled genome using BWA v. 0.6.2-r126 [92] using default parameters and calculating coverage on a panel of 140 single copy genes.

### Data access

RNASeq reads and transcriptome assemblies have been deposited at the NCBI Sequence Reads Archive (SRA) and NCBI Transcriptome shotgun assembly (TSA) databases respectively and are available under BioProject number PRJNA202292. Genomic reads and genome assembly have been deposited at the NCBI Sequence Reads Archive (SRA) and are available under BioProject number PRJNA202288. Assemblies have been deposited as Whole Genome Shotgun project at DDBJ/EMBL/GenBank under the accession ASRS00000000. The version described in this paper is version ASRS01000000.

## Competing interests

The authors declare that they have no competing interests.

## Authors’ contributions

MA initiated, designed the research work and wrote the manuscript; AM and AF performed the assembly and the annotation of the genome and the writing of the manuscript; MTV performed the extraction and purification of fungal and plant nucleic acids; PB performed phylogenetic analyses; LO and PT performed sequencing libraries preparation; GZ contributed to the bioinformatic data analysis; AI contributed to design the research work and cared the mycological part; GV and LC contributed to the design of the project and writing the manuscript; . MD designed the project and wrote the manuscript. All authors have read and approved the manuscript for publication.

## Supplementary Material

Additional file 1: Figure S1 Genome assembly statistics with Velvet at different k-mer length. A threshold 200 bp was set as the lowest accepted contig length. a) Number of assembled contigs. b) Maximum length of the assembled contigs. c) N50 of the assembly. d) Total sum of bases assembled in the contigs. **Figure S2.** Most Represented Species in Blast results. The chart reports, for the most represented species, the number of blast hits for *P. lycopersici* transcripts. **Figure S3.** Most Represented GO categories. The chart reports the number of the most represented GO categories among the assignments to *P. lycopersici* transcripts regarding: A) Process; B) Molecular Function. **Figure S4.** Comparison with *Fusarium oxysporum*. Homology regions at aminoacidic level are reported for each chromosome of *F. oxysporum*, in a vertical column, with colors representing the assembled contigs of *P. lycopersici*. Click here for file

Additional file 2: Table S1 Identified CEGMA ortholog genes. **Table S2.** Results of identification of major membrane transporter families domains in *P. lycopersici* and other 9 published transcriptomes. **Table S3.** Summary counts peptidase of homologs found in *P. lycopersici* transcriptome and in 9 published fungi transcriptomes. **Table S4.** Identification results of Heterokaryon Incompatibility proteins related domains in *P. lycopersici* (highlighted) and comparison with other fungal genomes. **Table S5.** Results of CAZyme domains identification comparison between *P. lycopersici* (highlighted) and other fungal genomes. **Table S6.** Carbohydrate-degrading enzymes in *P. lycopersici* (highlighted) and other Ascomycetes. Click here for file

Additional file 3 **Genome annotation.** This XLS document contains the annotation of *P. lycopersici* genome. Click here for file
