# Supplementary material for: De novo genome assembly of the soil-borne fungus and tomato pathogen Pyrenochaeta lycopersici
Source: BMC Genomics. 2014 Apr 27;15:313. doi: 10.1186/1471-2164-15-313 (PMC4234444; doi:10.1186/1471-2164-15-313)
Supplement: Additional file 1: Figure S1 — Genome assembly statistics with Velvet at different k-mer length. A threshold 200 bp was set as the lowest accepted contig length. a) Number of assembled contigs. b) Maximum length of the assembled contigs. c) N50 of the assembly. d) Total sum of bases assembled in the contigs. Figure S2. Most Represented Species in Blast results. The chart reports, for the most represented species, the number of blast hits for P. lycopersici transcripts. Figure S3. Most Represented GO categories. The chart reports the number of the most represented GO categories among the assignments to P. lycopersici transcripts regarding: A) Process; B) Molecular Function. Figure S4. Comparison with Fusarium oxysporum. Homology regions at aminoacidic level are reported for each chromosome of F. oxysporum, in a vertical column, with colors representing the assembled contigs of P. lycopersici. [file 1471-2164-15-313-S1.pdf]

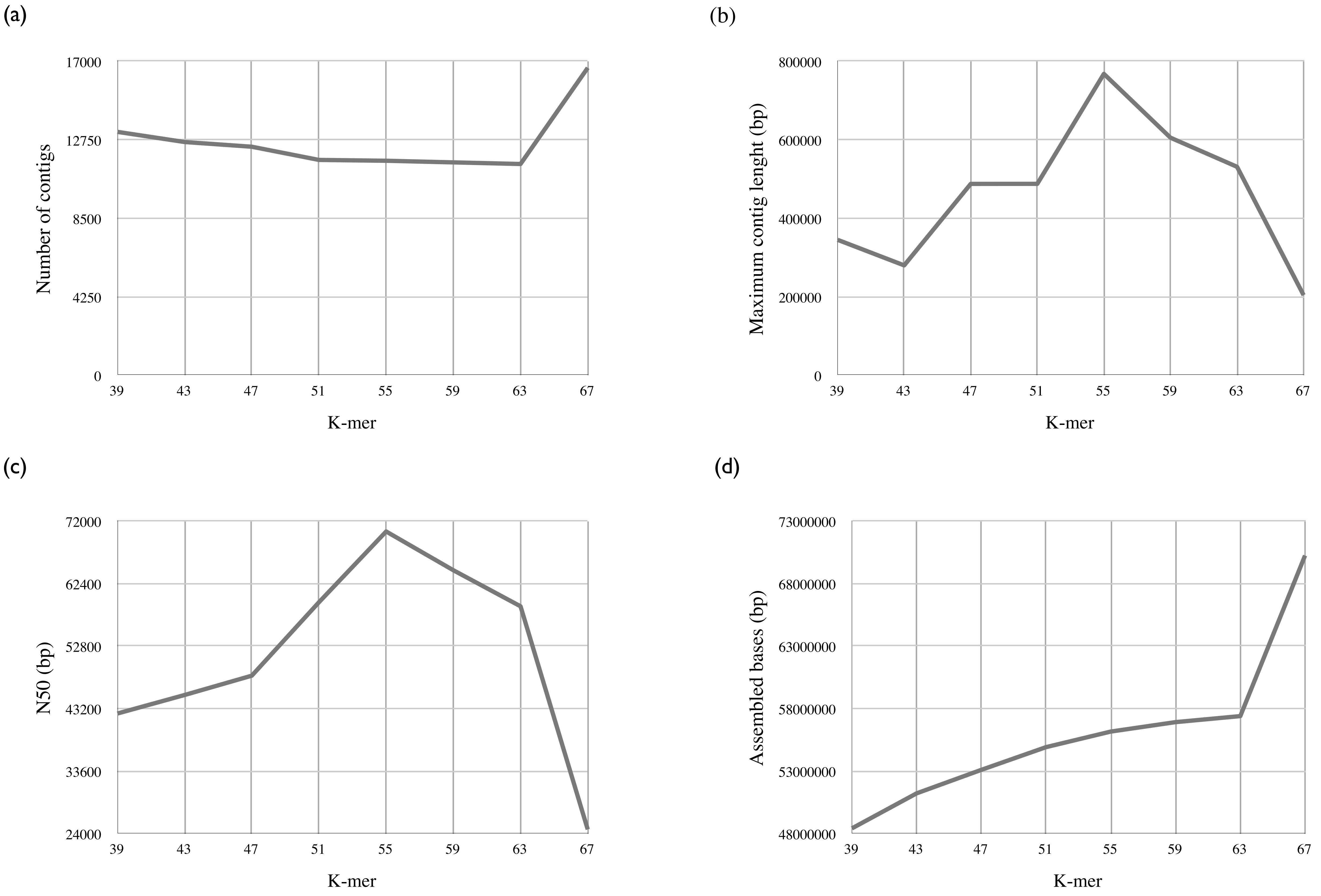

Figure S1: Genome assembly statistics with Velvet at different k-mer length. A threshold 200 bp was set as the lowest accepted contig length. a) Number of assembled contigs. b) Maximum length of the assembled contigs. c) N50 of the assembly. d) Total sum of bases assembled in the contigs.

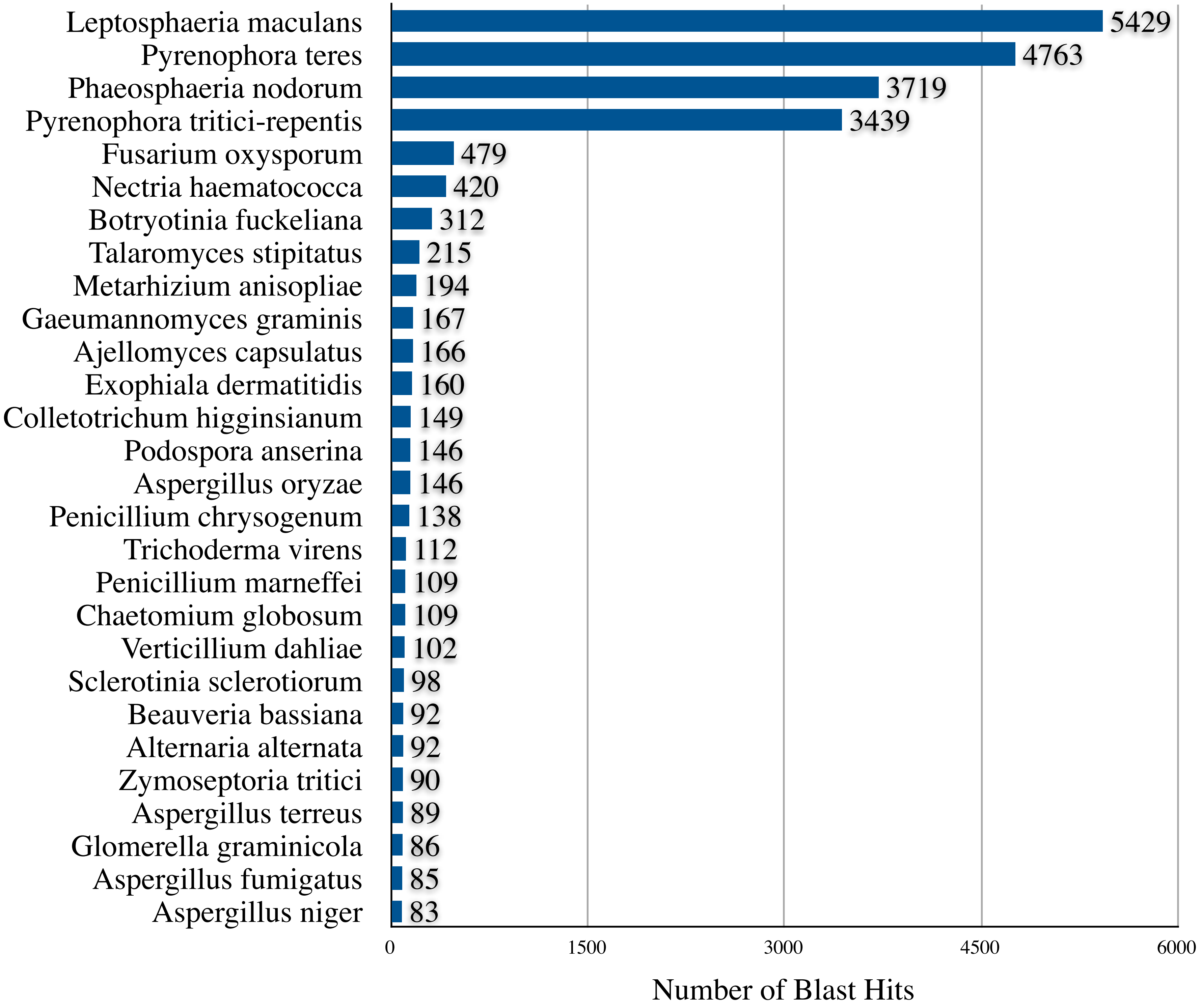

Figure S2. Most Represented Species in Blast results. The chart reports, for the most represented species, the number of blast hits for P. lycopersici transcripts.

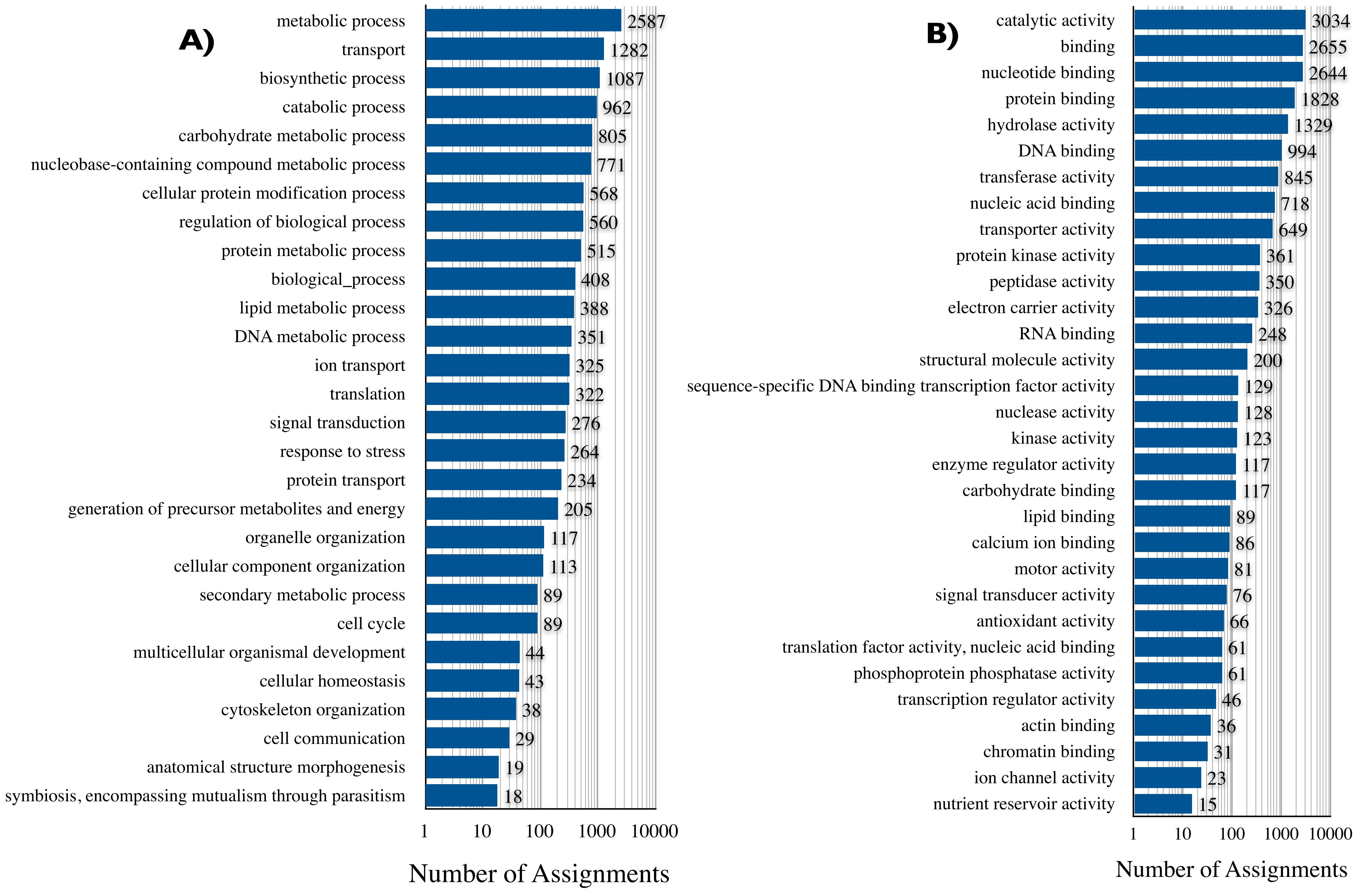

Figure S3. Most Represented GO categories. The chart reports the number of the most represented GO categories among the assignments to *P. lycopersici* transcripts regarding: A) Process; B) Molecular Function.

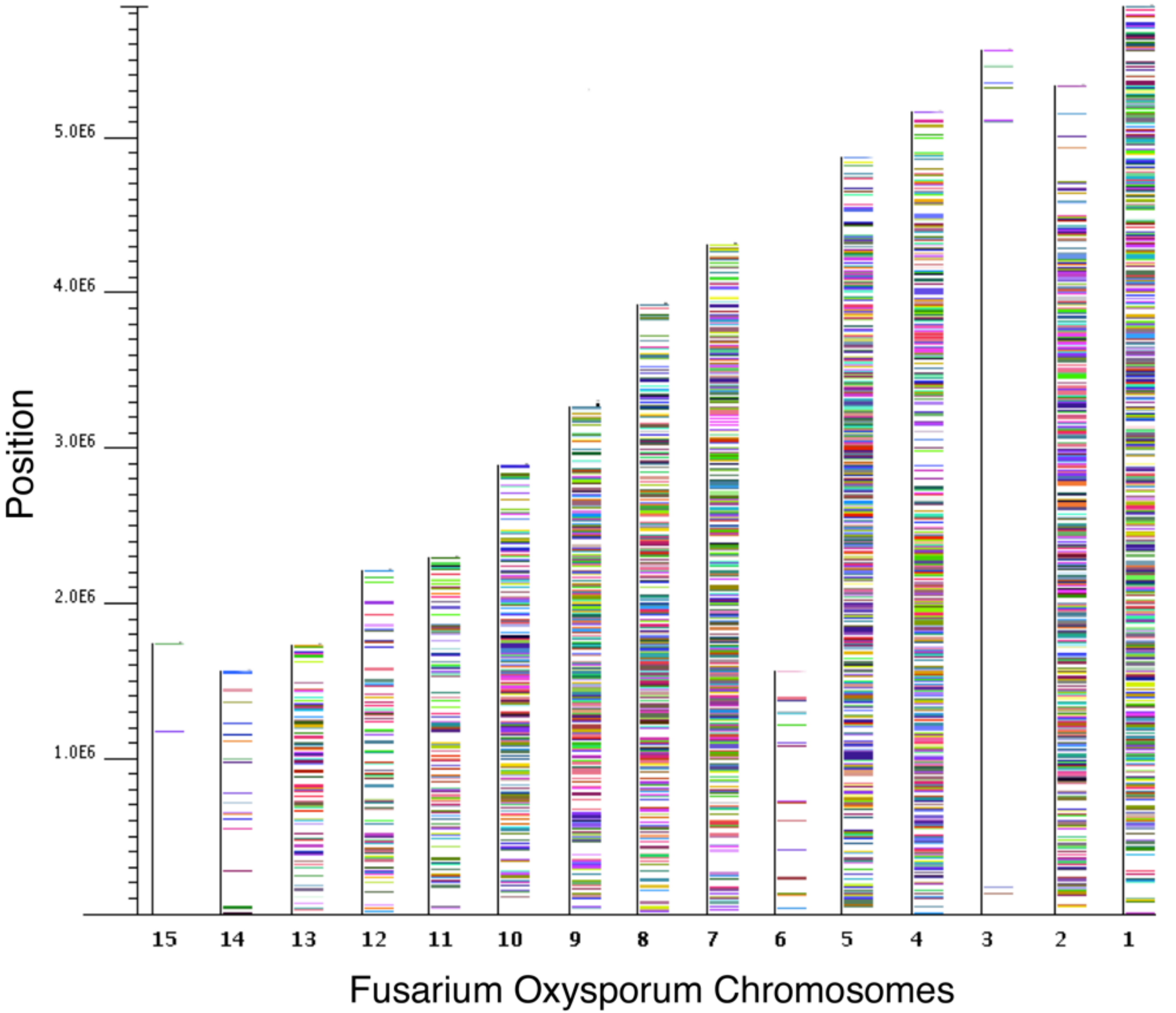

Figure S4. Comparison with Fusarium Oxysporum. Homology regions at aminoacidic level are reported for each chromosome of *F. oxysporum*, in a vertical column, with colors representing the assembled contigs of *P. lycopersici*.
